# Patterns and Predictors of Internet Gaming Disorder: An Observational Study from Jordan

**DOI:** 10.2174/1745017902117010217

**Published:** 2021-12-22

**Authors:** Reema Karasneh, Sayer Al-Azzam, Karem H. Alzoubi, Mohammad B. Nusair, Sahar Hawamdeh, Amal T. Nusir

**Affiliations:** 1 Department of Basic Medical Sciences, Faculty of Medicine, Yarmouk University, Irbid, Jordan; 2 Department of Clinical Pharmacy, Faculty of Pharmacy, Jordan University of Science and Technology, Irbid, Jordan; 3 Department of Pharmacy Practice and Pharmacotherapeutics, University of Sharjah, Sharjah, UAE; 4 Faculty of Pharmacy, Jordan University of Science and Technology, Irbid, Jordan; 5 Department of Clinical Pharmacy and Pharmacy Practice; Faculty of Pharmacy, Yarmouk University, Irbid , Jordan; 6 Department of Arabic Language and Literature, Faculty of Arts, Yarmouk University, Irbid, Jordan

**Keywords:** Gaming, IGD, Patterns, Mental disorders, Impairment, Addiction, Relapse, Psychosocial data

## Abstract

**Objective:**

Internet Gaming Disorder (IGD) is a rapidly growing public health problem that may have detrimental effects. The purpose of this study is to identify factors associated with IGD status.

**Methods:**

In this cross-sectional observational study, a convenient sample of gamers in Jordan was recruited and asked to participate in an online survey based on the nine criteria of the 20-item Internet Gaming Disorder (IGD-20) used to assess gaming disorder. Sociodemographic and psychosocial data were also obtained.

**Results:**

A total of 504 gamers participated in this study. The mean age of respondents was 21.6 ± 3.90 years. Using the standard IGD-20 scale, 96 participants (19%) were classified as potential IGD cases, compared to 408 (80.9%) non-disordered gamers. Males were dominant among the population, constituting 348 (69%) of gamers. Males also played significantly more hours per week [17.8 ±16.75] compared to females [13 ± 17.65]. The majority of gamers (411 (81.5%)) were students, although unemployed adults played for the highest total time [23.9 ± 30.84 hours/week]. Device type used for gaming also significantly (p <0.05) affected the time spent playing. Predictors of IGD included educational level (p< 0.05) and playing hours/week (p< 005). Conversely, no significant associations were found between IGD and gender, age, employment, or sleeping hours. IGD is increasingly being diagnosed among both genders and presents a health challenge for internet users.

**Conclusion:**

Establishing gamer profiles and recognizing predictors of IGD is therefore vital for guiding clinical classification and diagnosis of the disease.

## 1 INTRODUCTION

The technological advances of the last decades provided humans with a myriad of accessible and pleasurable gaming options. However, the introduction of internet gaming addiction in the Diagnostic and Statistical Manual of Mental Disorders, Fifth Edition (DSM-5) sparked controversy regarding the classification of certain gaming behaviours under 'non-substance addictive behaviours [1, 2].

Internet gaming disorder (IGD) the problematic use of computer games (whether online or offline)- has become a significant public health concern. Several studies have reported that IGD is comorbid with several other disorders and mental health issues, including impairment of personal and social functioning [2-4]. The DSM-5 has defined IGD as “persistent and recurrent use of the Internet to engage in games, often with other players, leading to clinically significant impairment or distress.” [5]. In addition, the WHO has defined gaming disorder in the latest beta draft version of the 11th revision of the International Classification of Diseases (ICD-11) as a new disorder [6]. After DSM-5 diagnosis of Internet Gaming Disorder (IGD), several studies have investigated its prevalence and factors that may be associated with its diagnosis and severity.

Variations in IGD prevalence rates were found among different populations and may be attributed to different tools used for IGD assessment and different cut-offs [7]. Several tools were developed for IGD assesment; however, the IGD-20 test was the first to assess IGD according to the nine criteria suggested by the American Psychiatric Association in the latest edition of the DSM-5 [8]. Worldwide, the prevalence of IGD ranged between 0.2% and 8.5%; the highest prevalence was estimated in the Korean population, with 50% of the population found to be addicted to gaming [9-11]. In a recent meta-analysis of 16 studies that investigated IGD prevalence among adolescents, the pooled prevalence of IGD was 4.6% (95% CI = 3.4%–6.0%) with higher prevalence rate reported in male adolescents (6.8%, 95% CI = 4.3%–9.7%) than female adolescents (1.3%, 95% CI = 0.6%–2.2%) [12]. Additionally, in a recent cross-sectional survey that has investigated IGD in European adolescents, 1.6% were diagnosed with IGD, with a further 5.1% being at risk for IGD. Among Europeans, IGD was highly associated with aggressive and rule-breaking behaviour and social problems [13]. Several factors were associated with IGD diagnosis, including male gender, age, poor self-esteem, and daily life satisfaction [13-16]. A recent systematic review by Mihara and Higuchi that included 37 cross-sectional and 13 longitudinal studies reported that IGD was associated with longer time spent playing games, higher frequency of playing games, more years playing games, and family and marital factors. Consequences related to IGD diagnosis included lower educational and career attainment (lower school grades, skipped school classes, and truancy), lower social skills, competence, and integration, in addition to higher impulsivity, neuroticism, aggression and violence [17].

In Jordan and the middle east area, studies are scarce about IGD. There is no account about IGD prevalence or profile of those with this disorder. Only a single study from the United Arab Emirates showed that IGD was associated with symptoms of mood disorder that worsen overtime. Thus, the current study came to establish a profile of disordered and non-disordered gamers and identifies the prevalence of IGD among adolescent and adult gamers. It also recognizes factors associated with an increased risk of IGD. Our study aims to and examines factors associated with IGD in the Jordanian population.

## 2 METHODS

### 2.1 Participants

Participants were recruited using online social media applications. All participants who completed the questionnaire were included.

### 2.2 Measures

The main questionnaire consisted of three sections: the first includes socio-demographic characteristics such as age, height, weight, sex, social status, education level, occupation, sleeping hours and the presence of chronic diseases. Respondents were inquired about their use of internet games within the preceding year. The second part inquired about the amount of daily play and used devices. Computer gaming behavior was assessed with a translated and validated Arabic version of the IGD-20 questionnaire [2].

IGD-20 Scale

The IGD-20 scale reflects the nine criteria of IGD, and it is based on the DSM-5 guidance and incorporates the theoretical framework of the components model of addiction [18]. Participants rated the 20 items on a 5-point Likert scale: 1 (“strongly disagree”), 2 (“disagree”), 3 (“neither agree nor disagree”), 4 (“agree”), and 5 (“strongly agree”). Participants were classified into two groups: disordered (Internet Gaming Disorder Group (IGDG; scoring 71 or more on the IGD-20)) and non-disordered or casual gaming group (CGG; scoring below 71 on the IGD-20) gamers. The use of a 71 cut-off point to diagnose IGD was proposed in an earlier study where it obtained adequate sensitivity and specificity [8].

### 2.3 Procedures

This is a descriptive, questionnaire-based study with a cross-sectional design. The questionnaire was distributed nationwide in Jordan using an online survey on social media based on voluntary participation. A brief description about the purpose of the study was provided.

### 2.4 Statistical Analysis

Statistical analyses comprised (i) descriptive statistical analysis of the main sample's characteristics, (ii) chi-square test, independent and paired samples student's t-test for mean comparisons for identifying possible significant group effects of the variables involved in this study on Internet usage and characteristics, and (iii) correlational analyses of the main variables included in the study using IBM SPSS Statistics Version 23.

### 2.5 Ethics

A brief description of the study was provided to participants prior to their participation. Participants were also informed prior to their participation that it would be completely anonymous, and no identifiable data will be obtained to reduce social desirability bias. Participation was completely voluntary and written informed consents were obtained from all study participants. Ethical approval for conducting the study was obtained from the institutional review board (reference code 32/119/2018). The study procedures were carried out in accordance with the Declaration of Helsinki.

## 3 RESULTS

Table **1** shows study participant demographics. A total of 504 participants enrolled in our study. Males were dominant 348 (69%) compared to females 156 (31%). The overwhelming majority of study subjects were students 411 (81.5%) with a mean age of 21.6 ± 3.90 years. Devices used for gaming varied between smartphones 380 (75.4%), a personal computer 177 (35.1%), a video game console such as PlayStation or Xbox 137 (27.2%), and tablets 38 (7.5%). Participants spent an average of 16.3 ± 17.28 hours per week playing (Table **1**).

There was a significant association between playing hours per week and gender (P value<0.005), as males were more likely to spend more hours playing than females. Employment status also affected total playing time, as unemployed gamers invested more time playing (23.9 ± 30.84 hrs/week playing) compared to students (16 ± 15.54 hrs/week) and employed gamers (13.4 ± 15.95 hrs/week). Using a computer compared to a tablet, smart phone, or gaming console was positively associated with more playing hours (P value < 0.041).

Difference was also assessed between disordered (n=96 (19%)), and non-disordered gamers (n=408 (80.9%)) (Table **2**). There were no significant differences between disordered and non-disordered gamers in age, body mass index, sleeping hours, gender, or marital status. However, playing hours/ week (p <0.005) and educational level (p< 0.05) were significantly higher among disorder gamers.

Participants completed an Arabic translated version of the IGD-20 tool (Table **3**), measuring six aspects of behavioural addiction: ‘Salience’, ‘Mood Modification’, ‘Tolerance’, and ‘Withdrawal Symptoms’, ‘Conflict’, and ‘Relapse’. Most gamers (44.1%, n= 222) denied losing sleep because of long gaming sessions. However, the majority (46.2%, n= 233) stated that gaming has become the most time-consuming activity in their daily life. Over half of the participants (52.5%, n= 263) said that they played games to help them cope with any bad feelings they might have or to forget about whatever is bothering them (52%, n= 262). On the contrary, around 38.7% (n=195) of the participants claimed they never play games to feel better.

About 22.4% (n=113) of gamers reported feeling irritable when not playing. Similarly, 21.5% (n=108) and 24.2% (n=122) of participants reported feeling sad or anxious if they were unable to play games, respectively. Most participants (42.6%, n= 215) agreed that gaming is negatively affecting important areas in their lives. Likewise, the majority of participants (43%, n= 222) believed that gaming has negative impacts on their daily activities, such as work, education, or household tasks. When asked if they believed they could stop gaming, most participants (49.4%, n= 249) agreed, while around 31.8% (n= 160) disagreed. About 30.7% also reported being unable to reduce gaming duration despite trying. In fact, over half of gamers (54.5%, n= 272) stated they have significantly increased the amount of gaming time over only the last year.

## 4 DISCUSSION

In this study, the majority of gamers were males, although there were no significant differences by gender in being a disordered or non-disordered player. Students constituted the majority of participants, although unemployed gamers played for more hours in total. Gender and device type were also factors associated with the number of hours played per week.

Disordered gamers comprised 96 out of our 504 players study population, making the prevalence of IGD among gamers around 19%. Comparably, prevalence rates previously reported in literature ranged from as low as 0.6% to as high as 8.5%, with higher rates in Asian countries and among male adolescents [2, 8]. However, one should bear in mind that most of these studies measured IGD in general populations such as university students and internet users. Therefore, the high prevalence reported in this study can be justified by the restriction of enrolment to gamers only. Indeed, a study from Singapore examining IGD among internet game players reported a similar percent of 17.7% disordered gamers [18]. The profile of IGD patients was also comparable to the one obtained in our study, as most disordered gamers were of primary to secondary education and were current students [18]. Another study with a 1420 gamers population showed a 3.6%-44% prevalence of addicted gamers [19]. In accordance with current results, time spent playing per week significantly correlated with online gaming addiction [20]. This was further confirmed by results from India, where time spent playing, in addition to greater depressive symptoms and a preference of multiplayer online gaming were all strongly predictive of gaming disorders [20]. A report from King *et al*. shows that as the time invested in playing increases, gamers receive more motivations for playing more, such as status among gamers and collecting game rewards [21]. This may justify the strong correlation between total gaming time and IGD; it especially reflects the tolerance aspect of the addiction. In fact, over half of gamers (54.5%) in our study stated they have significantly increased the amount of gaming time over only the last year. Other aspects reflecting addictive behaviour were also recognized among participants to varying extents, including withdrawal, conflict, and relapse.

In addition to total playing time per week, predictors of IGD in this study included educational level. Gamers acquiring up to secondary education were the most disordered, while post-graduates constituted only 8.3% of disordered gamers. This agrees with another study investigating IGD among international female gamers, where education was highly predictive of IGD. This study also showed correlation between the type of occupation and developing IGD, as unemployed gamers had the highest IGD scores [22]. Other studies have also reported higher IGD scores among those with more free time to invest in playing [23, 24]. However, the current study finds no significant association between IGD and occupation, with unemployed gamers constituting only 13% of disordered patients, despite investing more hours in gaming.

IGD is widely regarded as a 'man's disease', with a large study showing that 11.9% of males were diagnosed with IGD compared to 2.9% females [25]. These gender-based differences may be because more gamers in general are males. Indeed, in the current study, males constituted the majority of gamers 348 (69%). This may be due to several factors. Firstly, the design of online and video games predominantly draws attention and satisfies the needs of males in a male-dominant gaming industry [26]. Moreover, females do not conform to typical gamer stereotypes, often perceived asmasculine, leading to stigmatization of female gamers by the gaming community [27]. This is further perpetuated by the over-sexualization of female avatars, reflecting an objectification of women in these games [28]. Despite that, IGD is increasingly being diagnosed among females [1, 25]. In the current study, gender was associated with playing hours per week, as males were more likely to spend more hours playing than females (P value<0.005). However, no association was found between gender and addictive gaming. Two other studies agreed with these results, where gender was not a significant predictor of game addiction [9, 29]. Therefore, perception of IGD as a disease of males may limit diagnosis in females, who may show different characteristics of the disease [22, 30].

Devices used for gaming among participants, in the current study, varied between smartphones, a personal computer, a video game console such as PlayStation or Xbox, and tablets. Despite smartphones being the most used device for playing, those who played using computers spent the highest hours per week playing. A previous study has investigated gaming patterns across different devices [31]. Combined users of smartphones and computer games appeared to be more at risk of developing IGD and other psychological disorders. Meanwhile, the lowest prevalence of IGD was seen among smartphone users. Another study found a correlation between online games and IGD [32]. The study compared online and offline games among a sample of disordered and non-disordered players. Disordered players were found to spend three to four times as much time as non-disordered gamers playing online games.

The current study has a few limitations. Firstly, the questionnaire-based strategy may subject responses to recruitment and recall biases. Means to verify information regarding age and other demographics were also lacking due to online recruitment, creating a chance for misreports. Notably, the results of the current study were generally consistent with previous studies from other populations. Moreover, questionnaires represent the basis for most IGD studies, proving consistently as a successful method for this type of research. The relatively small sample size may also limit the ability of this study to detect differences in the prevalence of IGD between genders. Furthermore, the current study used convenient rather than random sampling as the study targeted internet gamers as its population, where means for taking a random sample from this group is not present as there is no official listing for internet gamers.

## CONCLUSION

Following the inclusion of internet gaming disorder (IGD) in the fifth edition of the Diagnostic and Statistical Manual of Mental Disorders (DSM-5), IGD became a subject of concern in internet game users. Since then, various studies have attempted to create a profile for gamers from both genders, and among different geographical areas. Our study shows a predominance of male gamers, albeit with no difference in game addiction compared to females. Total time spent gaming per week and educational level were the strongest predictors of IGD, while employment and device type predicted time spent playing. More research is recommended to understand disordered gamer profile in greater detail, especially in under-studies populations such as females.

## Figures and Tables

**Table 1:** Participants socio-demographic characteristics (n=504).

**Demographic Variables**		**Frequency (%)**	**Playing Hours/Week** **(mean ± SD)**	**P value**
**Gender**	Male	348 (69%)	17.8 ±16.75	<0.005
	Female	156 (31%)	13 ± 17.65	
**Education**	Up to secondary level	72 (14.3%)	19.3 ± 17.95	.274
	Bachelor’s degree or diploma	420 (83.3%)	15.9 ± 17.29	
	Post-graduate degree	12 (2.4%)	13.8 ± 10.89	
**Employment**	Employed	56 (11.1%)	13.4 ± 15.95	.012
	Unemployed	37 (7.3%)	23.9 ± 30.84	
	Student	411 (81.5%)	16 ± 15.54	
**Martial Status**	Single	484 (96%)	16.1 ± 16.21	.134
	Married	20 (4%)	22 ± 34.30	
**Chronic Conditions**	No	483 (95.8%)	16.4 ± 17.23	.700
	Yes	21 (4.2%)	14.9 ± 18.61	
**Device(s) used for gaming**	Smart phone	380 (75.4%)	15.8 ± 16.46	.041
	Computer	177 (35.1%)	20.1 ± 18.05	
	Tablet	38 (7.5%)	14.4 ± 16.22	
	Video game console (PlayStation, Xbox… *etc*.)	137 (27.2%)	16.2 ± 17.35	
**Age** (mean ± SD)			21.6 ± 3.90	
**Body Mass Index** (mean ± SD)			24.9 ± 8.81	
**Sleeping hours** (mean ± SD)			7.7 ± 1.75	
**Playing hours/week** (mean ± SD)			16.3 ± 17.28	

**Table 2:** Association between gaming disorder status and sociodemographic parameters.

-	**Disordered Gamers (n=96)** **Frequency (%)**	**Non-disordered Gamers (n=408)** **Frequency (%)**	**P value**
**Gender**	-	-	-
Male	62 (17.8%)	286 (82.2%)	0.326
Female	34 (21.8%)	122 (78.2%)	-
**Marital Status**	-	-	-
Single	94 (19.4%)	390 (80.6%)	0.393
Married	2 (10%)	18 (90%)	
**Education level**	-	-	-
Up to secondary level	21 (29.2%)	51 (70.8%)	0.044
Bachelor’s degree or diploma	74 (17.6%)	346 (82.4%)	-
Post-graduate	1 (8.3%)	11 (91.7%)	-
**Employment**	-	-	-
Student	79 (19.2%)	332 (80.8%)	0.622
Employed	12 (21.4%)	44 (78.6%)	-
Unemployed	5 (13.5%)	32 (86.5%)	-
**Age**	21.4±4.87	21.6 ± 3.65	0.731
**Body mass index**	24.9 ± 6.58	24.8 ± 9.27	0.979
**Sleeping hours**	8.0 ± 1.72	7.6 ± 1.75	0.080
**Playing hours/week**	22.6 ± 20.47	14.8 ± 16.09	<0.005

**Table 3:** Frequency of IGD-20 items among participants.

**Item**	**Strongly Agree**	**Agree**	**Neither agree or disagree**	**Disagree**	**Strongly Disagree**
I often lose sleep because of long gaming sessions.	28 (5.6%)	124 (24.6%)	130 (25.8%)	138 (27.4%)	84 (16.7%)
I usually think about my next gaming session when I am not playing	23 (4.6%)	141 (28%)	94 (18.7%)	149 (29.6%)	97 (19.2%)
I think gaming has become the most time-consuming activity in my life	64 (12.7%)	169 (33.5%)	62 (12.3%)	134 (26.6%)	75 (14.9%)
I play games to help me cope with any bad feelings I might have.	80 (15.9%)	183 (36.3%)	77 (15.3%)	91 (18.1%)	73 (14.5%)
I never play games in order to feel better.	56 (11.1%)	139 (27.6%)	149 (29.6%)	130 (25.8%)	30 (6.0%)
I play games to forget about whatever’s bothering me	77 (15.3%)	185 (36.7%)	75 (14.9%)	96 (19%)	71 (14.1%)
I have significantly increased the amount of time I play games over last year.	79 (15.7%)	193 (38.8%)	68 (13.5%)	96 (19%)	68 (13.5%)
I need to spend increasing amounts of time engaged in playing games	48 (9.5%)	177 (35.1%)	96 (19%)	133 (26.4%)	50 (9.9%)
I often think that a whole day is not enough to do everything I need to do in-game	43 (8.5%)	85 (16.9%)	74 (14.7%)	189 (37.5%)	113 (22.4%)
When I am not gaming, I feel more irritable	36 (7.1%)	77 (15.3%)	79 (15.7%)	171 (33.9%)	141 (28.0%)
I feel sad if I am not able to play games.	22 (4.4%)	86 (17.1%)	92 (18.3%)	175 (34.7%)	129 (25.6%)
I tend to get anxious if I can’t play games for any reason.	27 (5.4%)	95 (18.8%)	76 (15.1%)	172 (34.1%)	134 (26.6%)
I have lost interest in other hobbies because of my gaming	35 (6.9%)	142 (28.2%)	66 (13.1%)	159 (31.5%)	102 (20.2%)
I have lied to my family members because the amount of gaming I do	36 (7.1%)	100 (19.8%)	61 (12.1%)	166 (32.9%)	141 (28%)
I know my main daily activity (*i.e*., occupation, education, homemaker, *etc*.) has not been negatively affected by my gaming.	60 (11.9%)	148 (29.4%)	74 (14.7%)	162 (32.1%)	60 (11.9%)
I think my gaming has jeopardised the relationship with my partner	39 (7.7%)	96 (19.0%)	83 (16.5%)	161 (31.9%)	125 (24.8%)
I believe my gaming is negatively impacting on important areas of my life	49 (9.7%)	166 (32.9%)	85 (16.9%)	123 (24.4%)	81 (16.1%)
I often try to play games less but find I cannot.	58 (11.5%)	124 (24.6%)	101 (20%)	148 (29.4%)	73 (14.5%)
I do not think I could stop gaming.	31 (6.2%)	129 (25.6%)	95 (18.8%)	163 (32.3%)	86 (17.1%)
I often try to play games less but find I cannot.	36 (7.1%)	119 (23.6%)	93 (18.5%)	166 (32.9%)	90 (17.9%)

## Data Availability

The data supporting the findings of the article is available from corresponding author [S.A.-A] upon reasonable request.

## LIST OF ABBREVIATIONS

DSM-5: = Diagnostic and Statistical Manual of Mental Disorders Fifth Edition
IGD: = Internet Gaming Disorder
ICD-11: = International Classification of Diseases
MMORPGs: = Massively Multiplayer Online Role-Playing Games
DSM-IV-TR: = Diagnostic and Statistical Manual of Mental Disorder fourth revised edition
GIAD: = Goldberg Internet Addiction Disorder scale
ISS: = Internet Stress Scale
